# Longitudinal Associations Between Arts Engagement and Flourishing in Young Adults: A Fixed Effects Analysis of the Panel Study of Income Dynamics

**DOI:** 10.1007/s42761-022-00133-6

**Published:** 2022-10-11

**Authors:** Jessica K. Bone, Feifei Bu, Jill K. Sonke, Daisy Fancourt

**Affiliations:** 1 Research Department of Behavioural Science and Health, Institute of Epidemiology and Health Care, University College London, 1-19 Torrington Place, London, WC1E 7HB UK; 2 Center for Arts in Medicine, University of Florida, Gainesville, FL USA

**Keywords:** Adolescence, Emerging adults, Arts, Music, Theater, Flourishing, Wellbeing

## Abstract

There is growing evidence on the impact of arts engagement on flourishing. However, social gradients in arts engagement and flourishing may have led to an overestimation of this impact, and there is a lack of longitudinal research in young people. We aimed to test the longitudinal associations between arts engagement and flourishing in emerging adults, accounting for observed and unobserved individual characteristics. We included 3,333 participants aged 18–28 from the Transition into Adulthood Supplement of the Panel Study of Income Dynamics. We measured flourishing across emotional, psychological, and social wellbeing, and frequency of engagement in artistic, musical, or theatrical organized activities, biennially 2005–2019. We analyzed data using fixed effects regression and Arellano-Bond methods to control for bidirectional relationships. Increases in arts engagement were associated with increases in flourishing, before and after adjusting for time-varying confounders. This relationship was driven by enhanced psychological and social wellbeing. After controlling for bidirectionality, increases in arts engagement predicted subsequent improvements in flourishing and social wellbeing. In sensitivity analyses, residential area was a moderator; arts engagement was only associated with increased flourishing in metropolitan (and not non-metropolitan) areas. Increases in arts engagement are associated with enhanced flourishing within individuals, and these associations hold across many subgroups of the population. Those in non-metropolitan areas may have fewer opportunities for arts engagement. Future work must consider how funding can be distributed to ensure that the arts are accessible across communities and geographical areas, providing all young people with opportunities to experience their potential benefits.

**Supplementary Information:**

The online version contains supplementary material available at 10.1007/s42761-022-00133-6.

Flourishing is a state of positive mental health that involves both feeling good about, and functioning well in, individual and community life, as well as the absence of mental illness (Keyes, 2002). Although there has been debate about the definition of flourishing (Willen et al., 2022), it has been conceptualized as the combination of the two broad domains of hedonic and eudaimonic wellbeing (Keyes, 2002, 2006b; Stone & Mackie, 2013). Hedonic wellbeing, also referred to as emotional wellbeing, relates to happiness and the presence of positive feelings. Eudaimonic wellbeing relates to finding meaning and value in life as well as human potential that results in positive functioning when realized. This includes both psychological wellbeing (positive functioning in individual life) and social wellbeing (positive functioning in community life). Flourishing can thus be described as the presence of emotional, psychological, and social wellbeing (Keyes, 2002). Enabling this positive mental state, which is also referred to as subjective wellbeing, is arguably the end goal of all health-related research (Keyes, 2006a).

There is evidence that the arts (including participatory arts, visual arts and crafts, and cultural and heritage activities) might enable flourishing in adulthood (Shim et al., 2021). One theoretical model proposed that visual art, music, literature, and drama contribute to flourishing through five positive outcomes: allowing people to find meaning in life, experiencing new or elevated emotional states, aesthetic appreciation of beauty or skills, entertainment, and bonding with others (Lomas, 2016). Another model suggested that the arts enhance flourishing through four mechanisms: positive absorption in experiences, developing psychological processes (e.g., self-efficacy and autonomy), taking on social roles and identities within the community (socialization), and developing one’s character, values, and beliefs (Tay et al., 2018). However, much of the previous research on arts and flourishing has focused on older adults (e.g., Fancourt & Steptoe, 2018; Menec, 2003; Tymoszuk et al., 2019; Weinberg & Joseph, 2017). Given the different “social ecology” of psychological experience in these earlier years, findings cannot simply be extrapolated to younger people (Percy-Smith, 2007). Early adulthood is a prolonged period of social and economic change (Furstenberg, 2015), with profound implications for later development and health (Andrews et al., 2020; Shanahan, 2000), in which there has been an increasing focus on the importance of promoting flourishing (Keyes, 2006a).

Intervention studies have demonstrated that the arts can provide psychological support for young people (Fancourt et al., 2020). Reviews of research exploring community-based engagement have found that participating in activities such as music, dance, singing, drama, and visual arts can improve self-confidence, self-esteem, social skills, peer interactions, and sense of belonging in adolescence (Bungay & Vella-Burrows, 2013; Daykin et al., 2008; Zarobe & Bungay, 2017). Given this range of benefits, it is likely that arts engagement enhances flourishing overall, but there is very little research testing this. Additionally, intervention studies have been limited by small samples, short follow-up periods, and a focus on time-limited engagement in a specific activity rather than any ongoing ubiquitous arts engagement.

In qualitative studies, young people have reported that listening to music and attending music festivals enhanced several aspects of flourishing, including social wellbeing, purpose in life, autonomy, and personal growth (Packer & Ballantyne, 2011; Papinczak et al., 2015). There is also some evidence from observational studies of more ubiquitous engagement. For example, more time spent participating in organized out-of-school activities such as the arts (but also including sports and other activities) at ages 12 to 18 was associated with increased flourishing 6 years later (*n* = 1,115; Mahoney & Vest, 2012). In another study, arts participation was associated with improvements in self-esteem, meaning and purpose in life, life satisfaction, and peer relations 1 year later, which together indicate enhanced flourishing (*n* = 643; Mansour et al., 2016; Martin et al., 2013). However, these studies included relatively small samples, and the limited longitudinal evidence means it is unclear whether dynamic changes in arts engagement are associated with changes in flourishing over time. Additionally, research has not addressed the social gradient in arts engagement (Bone, Bu et al., 2021; Mak & Fancourt, 2021) and flourishing (Huppert, 2009); both are influenced by factors such as individual and community socioeconomic position, education, race/ethnicity, and gender. Arts engagement may represent a form of cultural capital that is obtained by young people with more material resources. For example, it has been proposed that education has an important role in developing knowledge of and appreciation for diverse forms of culture (Bourdieu, 1986). Previous evidence for associations between arts engagement and flourishing could therefore be a result of confounding and differences in cultural capital.

We aimed to test the longitudinal associations between arts engagement and flourishing in young adults. We explored whether changes in frequency of engagement in artistic, musical, or theatrical organized activities were associated with changes in overall flourishing, and the three domains of flourishing (emotional, psychological, and social wellbeing). We used fixed effects models to address confounding and differences in cultural capital. We then tested whether associations differed according to a range of demographic, socioeconomic, and health-related factors. We also investigated the direction of associations, testing whether arts engagement predicted changes in flourishing after accounting for previous levels of flourishing. We expected increased arts engagement to be associated with increases in both concurrent and subsequent flourishing, with these associations evident across all domains of flourishing.

## Method

### Sample

This study used data from the Transition into Adulthood Supplement (TAS) of the Panel Study of Income Dynamics (PSID). The PSID began in 1968 with a nationally representative sample of over 18,000 individuals living in 5,000 families in the USA and has now collected data for more than 50 years from over 82,000 individuals. As of 2019, more than 9,500 families are followed.

The TAS began in 2005 with waves every 2 years. Data are currently available to 2019. Broadly, the TAS includes children of PSID families aged 18 to 28 years. Two different approaches have been used for the TAS sample eligibility. First, from 2005 to 2015, members of the 1997 PSID Child Development Supplement (CDS; a cohort of children aged 0 to 12 years in 1997) who were aged 18 and over were eligible. Second, in 2017 and 2019, all PSID sample members aged 18–28 years were eligible to complete the TAS, regardless of whether they were members of the original CDS sample. In both approaches, eligible participants were members of families who completed the core PSID interview in the same year.

Overall, the TAS includes a nationally representative sample of young adults aged between 18 and 28, who entered the TAS between 2005 and 2019. Response rates were high across these waves of the TAS, ranging from 85 to 92% (Table S1). Due to the age restrictions on TAS eligibility, each individual participated in between one and six of the eight waves of the TAS. A total of 4,776 unique individuals completed the TAS. In this study, we limited our sample to those who completed measures of arts engagement, flourishing, and time-varying confounders at two or more waves of the TAS. This resulted in a final analytical sample of 3,333 young adults, who completed an average of 3.6 waves (range 2–6 waves; *n* = 11,872 total observations).

### Ethics Approval

All participants gave informed consent, and this study has Institutional Review Board approval from the University of Florida (IRB201901792) and ethical approval from University College London Research Ethics Committee (project 18839/001).

### Measures

#### Arts Engagement

Arts engagement was measured with the same two items in every wave of the TAS (biennially between 2005 and 2019). Firstly, participants were asked whether they had been involved in any organized activities related to art, music, or the theater in the last 12 months (yes, no). This could have included a range of arts activities, such as being part of a band, dance, or drama group, going on a group trip to a play or concert, or having any kind of tuition in the arts. These activities span both domains of arts engagement, from participatory (actively creating or being involved in the arts) to receptive activities (experiencing art that has been created as an audience member). Participants who reported being involved in these activities were then asked about the frequency of their engagement in the last 12 months, with six response options ranging from less than once a month to every day. Given the low number of participants reporting engagement at each level of frequency, and to enable comparison of different levels of arts engagement to no engagement, we combined responses to both questions into one variable. This measure categorized arts engagement as never (“no” response to the first question), monthly (less than once a month, at least once a month), weekly (once a week, several times a week), or daily (almost every day, every day).

#### Flourishing

Flourishing was measured in every wave of the TAS with a 14-item Languishing-Flourishing scale, also referred to as the Mental Health Continuum Short-Form (MHC-SF; Lamers et al., 2011). This scale asked participants to rate how often in the past month they had experienced 14 positive indicators of subjective wellbeing in three domains: emotional (three items), psychological (six items), and social (five items; Table S2). Emotional wellbeing includes feeling happy, satisfied, and interested in life, whereas psychological wellbeing consists of feelings of autonomy, mastery, personal growth, and positive relations with others. Social wellbeing does not relate to loneliness or social support but involves feeling like an integral part of a positive community, with items measuring social integration and contribution, acceptance of others, and perceiving that society is working in a positive way (social coherence and actualization; Lamers et al., 2011). Responses were made on a six-point scale ranging from never to every day. The average score on each domain was calculated (range 1–6) as well as the total flourishing score, which was the sum of scores across the three domains (range 1–18). Higher scores indicate better wellbeing. This scale was adapted from the Midlife in the United States study of adults (Keyes, 2006b; Panel Study of Income Dynamics, 2010; Stafford et al., 2005) and the reliability and validity of the three factors (emotional, psychological, and social wellbeing) has been demonstrated in adults (Lamers et al., 2011) and young people (Keyes, 2006b). The internal consistency of the overall flourishing scale was good across waves (Cronbach’s *α* range 0.86–0.90), as was the internal consistency of each domain (emotional *α* = 0.78–0.87; psychological *α* = 0.79–0.85; social *α* = 0.74–0.80).

#### Demographic, Socioeconomic, and Health-Related Factors

We included a range of time-varying demographic, socioeconomic, and health-related factors. These were age (years), marital status (unmarried [never married, separated, widowed, divorced], married [including cohabiting]), level of education (high school or less, some college, college graduate), employment status (employed, unemployed, student), total family income (U.S. dollars), participants’ rating of their general health status (good to excellent, poor to fair), and whether a health professional has ever told participants that they have an emotional, nervous, or psychiatric problem (yes, no).

We also included time-invariant characteristics measured at each participant’s baseline (the first year they completed the TAS). Demographic factors were gender (male, female), race/ethnicity (White, Black [including African American, Negro], Other [including American Indian, Alaskan Native, Asian, Native Hawaiian, Pacific Islander, Hispanic]), and residential area of the family home (metropolitan, non-metropolitan). We measured socioeconomic position (SEP) as the sum of participant education tertiles, family income quartiles, and parental education tertiles. Finally, we included a score indicating the quality of the childhood home environment, as measured on the physical environment subscale of the interviewer-completed Home Observation for Measurement of the Environment (HOME) Inventory in the CDS in 2002 (Caldwell & Bradley, 1979; Panel Study of Income Dynamics, 2010). The total possible score ranged from 13 to 65, with higher scores indicating a better home environment.

### Statistical Analysis

Descriptive statistics are presented for all time-varying and time-invariant factors at baseline. Given that participants entered the TAS in different years, we took the baseline as the first wave at which each participant in the sample completed the TAS. The baseline therefore does not refer to a single wave of the TAS, but collapses across participants’ first completion of the TAS.

We used fixed effects regression to test the longitudinal associations between arts engagement and flourishing. This approach uses only within-individual variation to examine how the change in arts engagement is related to the change in flourishing within individuals over time. As individuals are compared with themselves over time, all time-invariant factors are accounted for automatically, even if unobserved. Fixed effects models thus control for individual heterogeneity, eliminating potential biases in the estimates of time-variant variables (Allison, 2009). We tested four fixed effects models, using flourishing, emotional wellbeing, psychological wellbeing, and social wellbeing as separate outcomes. We present all four models before and after adjustment for time-varying confounders (age, marital status, education, employment, family income, general health, emotional/psychiatric problem).

Fixed effects models do not assess the directionality of the association between arts engagement and flourishing so, to explore this, we adjusted for previous levels of flourishing. However, adjusting for previous flourishing introduces bias into fixed effects models because the previous values of the outcome are correlated with the error term of the model, meaning estimates are biased. To address this problem, we employed the Arellano-Bond approach, which uses a first-difference model and includes lags of the outcome variable as instruments for the first difference (Arellano & Bond, 1991). The advantage of this approach is that it takes account of previous changes in flourishing over time to estimate the effect of arts engagement on subsequent changes in flourishing, while accounting for differences in individual characteristics. Under the assumption that the error terms are not serially correlated across waves, which we tested using the Arellano-Bond test, the lagged outcomes are unrelated to the error term in the first difference and consistent estimates can be produced. We treated the exposure (arts engagement) and time-varying confounders as endogenous, using their values at previous waves as instruments. We also included an exogenous dummy variable for wave to improve the model estimation. We tested the joint validity of all instruments with the Sargan-Hansen test. We used two-step efficient generalized method of moments (GMM) with the system GMM estimator, the Windmeijer correction applied (Windmeijer, 2005), and included all available lags in the models. This approach maximized the sample size available for these models (Roodman, 2009).

All analyses were performed using Stata 17 (StataCorp, 2021) and Arellano–Bond models were fitted with the user-written command “xtabond2” (Roodman, 2009).

#### Moderation by Sociodemographic and Health-Related Factors

We tested whether the association between arts engagement and flourishing was moderated by a range of sociodemographic and health-related factors, namely age, gender, race/ethnicity, residential area of the family home, overall SEP, participant education, family income, parental education, childhood home environment, general health status, and emotional/psychiatric problems. To do this, we entered these time-invariant factors (measured at baseline or earlier) into the fixed effects models in interaction terms with arts engagement. This allowed us to test whether the association between changes in engagement and changes in flourishing differed according to these factors. Given that not all participants were still living in the family home, we also performed a sensitivity analysis limited to participants who reported living at their family home in either the fall/winter or summer of each wave. This tested whether the evidence for an interaction between residential area and arts engagement on flourishing was maintained in this subsample.

#### Sensitivity Analyses

Given the sensitivity of Arellano–Bond models to specification decisions (Roodman, 2007), we tested whether results from the Arellano–Bond models were robust to two alternate model specifications. First, we used the difference GMM estimator in two-step efficient GMM Arellano-Bond models instead of the system GMM estimator. Second, we retained all specifications of the original Arellano-Bond model but limited the number of included lags used for the outcome, exposure, and time-varying confounder instruments to two per participant.

## Results

The sample included 3,333 young adults who were an average age of 19.04 years (SD = 1.70) at baseline, of whom 52% were female, 48% were White, 43% were Black, 88% were unmarried, and 65% had some college education (Table 1). At baseline, 74% of participants reported never engaging in organized activities related to art, music, or the theater in the last 12 months. In contrast, 9% engaged monthly, 11% weekly, and 6% daily. Across the study period, 39% of participants reported engaging in the arts at one or more time point and 32% of participants reported changes in their level of arts engagement. There were also substantial changes in flourishing over time, as within-individual variation accounted for approximately 57% of the overall variation in flourishing, 51% in emotional wellbeing, 50% in psychological wellbeing, and 53% in social wellbeing (Table S3). At baseline, levels of flourishing differed across the different frequencies of arts engagement (Table S4).

**Table 1:** Demographic characteristics of the sample at baseline

	**Mean (SD)**
Age	19.04 (1.70)
Family income	$67,569.44 ($74,647.87)
HOME inventory score	56.30 (8.24)
	**Proportion**
Gender	
Male	48%
Female	52%
Race/ethnicity	
White	48%
Black	43%
Other	9%
Marital status	
Unmarried	88%
Married/cohabiting	12%
Education	
High school or less	33%
Some college	65%
College graduate	2%
Employment	
Employed	48%
Unemployed	21%
Student	31%
Residential area	
Metropolitan	70%
Non-metropolitan	30%
General health	
Good/excellent	91%
Poor/fair	9%
Emotional/psychiatric problem	
None	89%
One or more	11%
Parental education	
High school or less	40%
Some college	29%
College graduate	31%

*Note. N* = 3,333. All observed and unobserved time-invariant demographic characteristics are automatically accounted for in fixed effects models but are shown here for descriptive purposes. Baseline indicates the first wave at which each participant in the sample completed the TAS, and therefore does not relate to a single year of data. Participants were missing data in HOME inventory (38%), race/ethnicity (< 1%), residential area (< 1%), and parental education (< 1%)

### Flourishing

Increases in the frequency of arts engagement were associated with increases in flourishing (Table 2). This was a dose-response relationship: there was very little evidence that changing from no engagement to monthly engagement was associated with flourishing (coefficient [coef] = 0.13, 95% confidence interval [CI] = −0.03–0.28), but there was strong evidence that changing to both weekly (coef = 0.31, 95% CI = 0.16–0.47) and daily (coef = 0.52, 95% CI = 0.29–0.75) engagement were associated with increases in flourishing. After adjusting for time-varying confounders, there was still evidence that increasing from no engagement to weekly engagement was associated with a 0.28-point (95% CI = 0.13–0.44) increase in flourishing, which was equivalent to a 2.09% increase in flourishing. Similarly, changing from no engagement to daily engagement was associated with a 0.45-point (95% CI = 0.22–0.68) increase in flourishing, equivalent to a 3.34% increase.

**Table 2:** Fixed effects models testing the longitudinal associations between arts engagement and flourishing and the three domains of flourishing (emotional, psychological, and social wellbeing)

Arts engagement	Unadjusted		Adjusted^a^	
	Coef (95% CI)	*p* value	Coef (95% CI)	*p* value
Model 1: flourishing				
Monthly	0.13 (−0.03 to 0.28)	0.103	0.12 (−0.03 to 0.28)	0.108
Weekly	**0.31 (0.16 to 0.47)**	**< 0.001**	**0.28 (0.13 to 0.44)**	**< 0.001**
Daily	**0.52 (0.29 to 0.75)**	**< 0.001**	**0.45 (0.22 to 0.68)**	**< 0.001**
Model 2: emotional wellbeing				
Monthly	0.02 (−0.05 to 0.08)	0.597	0.01 (−0.05 to 0.08)	0.672
Weekly	**0.08 (0.02 to 0.14)**	**0.013**	**0.06 (0.00 to 0.13)**	**0.045**
Daily	0.08 (−0.01 to 0.17)	0.089	0.05 (−0.04 to 0.14)	0.300
Model 3: psychological wellbeing				
Monthly	0.04 (−0.02 to 0.10)	0.185	0.03 (−0.02 to 0.09)	0.255
Weekly	**0.08 (0.02 to 0.14)**	**0.009**	**0.06 (0.00 to 0.12)**	**0.050**
Daily	**0.18 (0.09 to 0.27)**	**< 0.001**	**0.14 (0.04 to 0.23)**	**0.004**
Model 4: social wellbeing				
Monthly	0.04 (−0.04 to 0.12)	0.329	0.04 (−0.03 to 0.12)	0.277
Weekly	**0.16 (0.08 to 0.24)**	**< 0.001**	**0.16 (0.08 to 0.24)**	**< 0.001**
Daily	**0.23 (0.11 to 0.34)**	**< 0.001**	**0.22 (0.10 to 0.33)**	**< 0.001**

*Note*. *N* = 3,333 (11,872 observations). Reference category in all models was never. Bold text indicates *p* < 0.05

^a^Time-varying confounders were age, marital status, education, employment, family income, general health, and emotional/psychiatric problems

When testing the direction of this relationship (Table 3), there was initially very weak evidence that daily arts engagement predicted subsequent increases in flourishing (unadjusted coef = 1.23, 95% CI = −0.01–2.47). Adjusting for time-varying confounders increased the evidence for this association as, in the fully adjusted model, both weekly (coef = 0.76, 95% CI = 0.02–1.50) and daily (coef = 1.26, 95% CI = 0.25–2.27) arts engagement predicted subsequent increases in flourishing. In this model, the change in flourishing for increasing from no to weekly engagement was equivalent to a 5.61% increase and daily engagement was equivalent to a 9.31% increase in flourishing.

**Table 3:** Arellano-Bond models testing whether arts engagement predicted subsequent flourishing and the three domains of flourishing (emotional, psychological, and social wellbeing)

Arts engagement	Unadjusted		Adjusted^a^	
	Coef (95% CI)	*p* value	Coef (95% CI)	*p* value
Model 1: flourishing				
Monthly	0.61 (−0.65 to 1.87)	0.341	0.08 (−0.66 to 0.82)	0.839
Weekly	0.95 (−0.18 to 2.08)	0.099	**0.76 (0.02 to 1.50)**	**0.045**
Daily	1.23 (−0.01 to 2.47)	0.053	**1.26 (0.25 to 2.27)**	**0.014**
Model 2: emotional wellbeing				
Monthly	−0.15 (−0.69 to 0.39)	0.588	0.00 (−0.30 to 0.31)	0.980
Weekly	0.23 (−0.22 to 0.68)	0.318	0.15 (−0.12 to 0.43)	0.267
Daily	−0.08 (−0.56 to 0.41)	0.761	0.00 (−0.39 to 0.39)	0.998
Model 3: psychological wellbeing				
Monthly	0.10 (−0.39 to 0.58)	0.697	0.01 (−0.29 to 0.31)	0.930
Weekly	0.08 (−0.35 to 0.52)	0.706	0.11 (−0.15 to 0.37)	0.390
Daily	0.36 (−0.11 to 0.83)	0.134	0.33 (−0.12 to 0.78)	0.146
Model 4: social wellbeing				
Monthly	**0.58 (0.01 to 1.14)**	**0.045**	0.19 (−0.16 to 0.53)	0.289
Weekly	0.59 (−0.03 to 1.21)	0.063	0.32 (−0.10 to 0.74)	0.139
Daily	**1.03 (0.32 to 1.73)**	**0.004**	**0.80 (0.15 to 1.44)**	**0.015**

Note. *N* = 3,175 (7,955 observations). Reference category in all models was never. Bold text indicates *p* < 0.05

^a^Time-varying confounders were age, marital status, education, employment, family income, general health, and emotional/psychiatric problems

### Domains of Flourishing

#### Emotional Wellbeing

There was very little evidence that increases in arts engagement were associated with increases in emotional wellbeing (Table 2). Before and after adjusting for time-varying confounders, weekly (but not daily) engagement was associated with a small increase in emotional wellbeing (adjusted coef = 0.06, 95% CI = 0.00–0.13; equivalent to a change of 1.30%).

When looking at the direction of this relationship (Table 3), there was no evidence that changes in arts engagement predicted subsequent changes in emotional wellbeing.

#### Psychological Wellbeing

There was some evidence for a longitudinal association between arts engagement and psychological wellbeing (Table 2). In the fully adjusted model, changing from no engagement to weekly arts engagement was associated with a 0.06-point (95% CI = 0.00–0.12) improvement in psychological wellbeing, and changing to daily arts engagement was associated with a 0.14-point (95% CI = 0.04–0.23) increase in psychological wellbeing. In this model, the change in wellbeing for increasing from no to weekly engagement was equivalent to a 1.23% increase and daily engagement was equivalent to a 2.71% increase in psychological wellbeing.

When examining the direction of this relationship (Table 3), there was no evidence that changes in arts engagement predicted subsequent changes in psychological wellbeing.

#### Social Wellbeing

The strongest evidence was for the longitudinal association of arts engagement with social wellbeing (Table 2). After adjusting for time-varying confounders, increasing from no to weekly arts engagement was associated with a 0.16-point (95% CI = 0.08–0.24) improvement in social wellbeing, and changing to daily arts engagement was associated with a 0.22-point (95% CI = 0.10–0.33) increase in social wellbeing. In this model, the change in wellbeing for increasing from no to weekly engagement was equivalent to a 4.50% increase and daily engagement was equivalent to a 6.16% increase in social wellbeing.

When investigating the direction of this relationship (Table 3), there was initially weak evidence that monthly (coef = 0.58, 95% CI = 0.01–1.14) and daily arts engagement (coef = 1.03, 95% CI = 0.32–1.73), and to some extent also weekly arts engagement (coef = 0.59, 95% CI = −0.03–1.21), predicted subsequent increases in social wellbeing. After adjusting for time-varying confounders, this evidence was attenuated, and only daily arts engagement predicted subsequent increases in social wellbeing (coef = 0.80, 95% CI = 0.15–1.44). This was equivalent to a 22.94% increase in social wellbeing.

### Moderation by Sociodemographic and Health-Related Factors

Testing moderators in the fixed effects models, there was no evidence that age, gender, race/ethnicity, overall SEP, the socioeconomic factors separately (participant education, family income, and parental education), childhood home environment, general health status, or having an emotional or psychiatric problem at baseline moderated the association between changes in arts engagement and flourishing (Table 4). However, there was evidence that the longitudinal association between arts engagement and flourishing differed according to whether participants lived in a metropolitan area (interaction coef = −0.17, 95% CI = −0.30 to −0.04). In non-metropolitan (rural) areas, there was no evidence for an association between arts engagement and flourishing (Table S5). However, in metropolitan areas, there was a strong dose-response relationship, even after adjusting for time-varying confounders; increasing levels of arts engagement were associated with larger improvements in flourishing compared to no engagement, from monthly (coef = 0.19, 95% CI = 0.01–0.37), to weekly (coef = 0.41, 95% CI = 0.22–0.60), to daily (coef = 0.55, 95% CI = 0.27–0.83) engagement.

**Table 4:** Interaction terms from the fixed effects models testing whether demographic, socioeconomic, and health-related factors moderated the longitudinal associations between arts engagement and flourishing

Moderator	Interaction term	
	Coef (95% CI)	*p* value
Age	0.03 (−0.03 to 0.08)	0.279
Gender	0.08 (−0.03 to 0.20)	0.163
Race/ethnicity: Black	0.01 (−0.11 to 0.14)	0.867
Race/ethnicity: Other	0.10 (−0.12 to 0.32)	0.357
Residential area	**−0.17 (−0.30 to −0.04)**	**0.009**
Socioeconomic position	0.02 (−0.02 to 0.05)	0.312
Education	0.09 (−0.05 to 0.24)	0.190
Family income	0.02 (−0.04 to 0.08)	0.465
Parental education	0.03 (−0.04 to 0.10)	0.429
Childhood home environment	0.00 (−0.01 to 0.01)	0.508
General health status	0.12 (−0.17 to 0.40)	0.420
Emotional/psychiatric problem	0.00 (−0.19 to 0.20)	0.971

*Note. N* = 3,333 (11,872 observations), except for race/ethnicity (*N* = 3,331; 11,866 observations), residential area (*N* = 3,328; 11,860 observations), and parental education (*N* = 3,317; 11,824 observations). All models adjusted for time-varying confounders (age, marital status, education, employment, family income, general health, and emotional/psychiatric problems). Bold text indicates *p* < 0.05

After limiting the sample to participants who reported living at their family home in either the fall/winter or summer of each wave (*n* = 6,425), the evidence for this interaction was attenuated (interaction coef = −0.10, 95% CI = −0.30 to 0.10). Nevertheless, examining the residential area strata separately, findings were similar to the whole sample. In non-metropolitan (rural) areas, there was no evidence for a longitudinal association between arts engagement and flourishing (Table S6). In contrast, in metropolitan areas, there was evidence that monthly (coef = 0.29, 95% CI = 0.01–0.59) and weekly (coef = 0.33, 95% CI = 0.05–0.61) arts engagement was associated with increased flourishing. Although this evidence was attenuated after adjusting for time-varying confounders, the association with weekly engagement remained significant (coef = 0.31, 95% CI = 0.03–0.58).

### Sensitivity Analyses

Our findings on the direction of associations between arts engagement, flourishing, and the domains of flourishing were generally robust to the specification of the Arellano-Bond models. Using the difference GMM estimator (instead of the system GMM estimator) greatly reduced the available sample size. In this model, only daily arts engagement predicted subsequent flourishing and social wellbeing, and this relationship was attenuated after including time-varying confounders (Table S7). However, estimates were similar to, and within the confidence intervals of, the results of the main analyses. In a second sensitivity analysis, limiting the number of lags included in the Arellano-Bond models to two per participant also led to similar estimates but with wider confidence intervals (Table S8). In both the unadjusted and adjusted models, there was only evidence that daily arts engagement predicted subsequent social wellbeing.

## Discussion

In this study, most young people (61%) never engaged in organized activities related to art, music, or the theater. However, for those who did participate, increasing time spent doing organized activities related to art, music, or the theater was associated with enhanced flourishing. This was a dose-response relationship, as engaging in the arts daily was associated with larger increases in flourishing than engaging weekly, which was associated with larger increases than engaging monthly. The association with flourishing was driven primarily by increases in social wellbeing (feeling like an integral part of a positive community), accompanied by smaller increases in psychological wellbeing (feelings of autonomy, mastery, and personal growth). In contrast, there was very little evidence that arts engagement was associated with emotional wellbeing (feeling happy, satisfied, and interested in life). Looking at the direction of these relationships, increases in arts engagement predicted subsequent increases in flourishing, which were also driven by increases in social wellbeing.

Although monthly arts engagement may not be sufficient to enhance flourishing, more frequent engagement appears to enhance flourishing by improving social wellbeing in young people. Within this flourishing framework, social wellbeing does not relate to loneliness or social support but involves feeling like an integral part of a positive community (Lamers et al., 2011). Young people who engage in the arts more frequently are thus more likely to see their own daily activities as useful to and valued by others, be interested in their social life and find it meaningful, feel a sense of belonging to as well as comfort and support from their community, have positive attitudes towards and be accepting of human differences, and believe that people and society have potential to evolve or grow positively (Keyes, 2006b). Enhancing social wellbeing is important during early adulthood, a prolonged period of social disruption and development (Furstenberg, 2015), with profound implications for later development and health (Andrews et al., 2020; Shanahan, 2000). Additionally, in line with the trends observed in this study, previous research has found that social wellbeing is lower than emotional and psychological wellbeing in young people in the USA (Keyes, 2006b). Activities that can enhance social wellbeing, such as arts engagement, may thus be particularly beneficial for young people.

This finding is consistent with models proposing that arts promote flourishing through bonding with others (Lomas, 2016) and socialization (Tay et al., 2018). It is also consistent with evidence that arts engagement can improve mental health through varied social mechanisms (Fancourt et al., 2021). Arts activities are likely to provide young people with a community of likeminded peers, help them feel that they have something to contribute, and improve social skills and confidence. Additionally, arts activities may provide resources that can be used to develop new social identities (Dadswell et al., 2017). According to social identity theory, individuals build a sense of who they are based on their group membership (Hogg, 2006), and those with stronger social identities have enhanced wellbeing (Haslam et al., 2009). We have previously found that engaging in more extracurricular arts activities is associated with higher levels of social support during adolescence (Bone, Fancourt et al., 2021). Similarly, music, dance, and theater interventions promote social connectedness and improve relationships for young adults (Bradley et al., 2004; Gardner et al., 2008; Koch et al., 2015; Papinczak et al., 2015).

Arts engagement did not predict subsequent psychological wellbeing, but weekly or daily engagement was associated with concurrent increases in this domain. A dynamic relationship thus exists independent of the broader aspects of social and cultural capital and socioeconomic position that differ across individuals and influence arts engagement (Bone, Bu et al., 2021; Mak & Fancourt, 2021) and wellbeing (Huppert, 2009). This is consistent with proposals that arts engagement may contribute to a sense of purpose and meaning in life, aid character development, and support the development of psychological processes such as autonomy (Lomas, 2016; Ryff & Kim, 2020; Tay et al., 2018). The relationship between arts engagement and psychological wellbeing may be bidirectional. Young people with higher psychological wellbeing feel more able to achieve their goals so may be more likely to participate in arts activities. Consistent with this, older adults with higher psychological wellbeing are more likely to engage in arts and cultural activities (Steptoe & Fancourt, 2019).

The inconsistent association between arts engagement and emotional wellbeing was unexpected. There is extensive evidence that arts engagement is associated with increased positive affect and life satisfaction (Mansour et al., 2016; Martin et al., 2013), although most of it is from studies of older adults (Bone et al., 2022; Fancourt & Steptoe, 2018; Lakey et al., 2017; Wang et al., 2020; Wheatley & Bickerton, 2017). It is possible that factors contributing to emotional wellbeing differ in early and later adulthood. Additionally, prior findings in young people may be a result of confounding by demographic and socioeconomic factors. This could also explain why our findings on arts engagement and subsequent psychological wellbeing differ to previous longitudinal evidence (Mansour et al., 2016; Martin et al., 2013).

Our findings held across many subgroups of the population, indicating that all young people have the potential to benefit from the arts. Despite the relatively low rates of arts engagement in our sample, and the possibility that young people with more cultural capital were more likely to be engaging (Bone, Bu et al., 2021; Mak & Fancourt, 2021), we found no evidence that overall SEP or separate socioeconomic factors (participant education, family income, and parental education) moderated the association between arts engagement and flourishing. However, arts engagement was not associated with flourishing in non-metropolitan areas. These individuals may be affected by the presence of fewer opportunities for arts engagement (Bone, Bu et al., 2021; Mak & Fancourt, 2021). Alternatively, this finding may be linked to community-level socioeconomic position, as arts engagement is more strongly associated with enhanced wellbeing in communities with lower socioeconomic position (Mak, Coulter & Fancourt, 2021), many of which are metropolitan in the USA (Kneebone, 2014). Future work must consider how funding can be distributed to ensure that the arts are accessible across communities and geographical areas, providing all young people with opportunities to experience their potential benefits.

Understanding whether arts engagement has benefits for long-term flourishing is key in the current policy landscape. Our findings demonstrate the importance of ensuring young people can engage in organized arts activities. However, the U.S. government has repeatedly proposed cutting all federal arts funding, and there are frequent debates about the extent to which the arts should be part of educational curricula (e.g., McGlone, 2020). When adjusted for inflation, funding for the arts in education has decreased by 30% over the past 20 years (Jung, 2018). Given the time that young people spend in education, as well as barriers to and increased social gradients in out-of-school arts activities (Mak & Fancourt, 2021), arts programs and policies that ensure funding for arts are key. This is particularly important alongside our finding that 61% of young people never reported engaging in organized arts activities during the study period. Work is currently underway in countries such as the UK, as well as in pilots in the USA, to bring arts to young people via social prescribing (SP) schemes. SP involves referring young people to a link worker, who connects them to activities like the arts with the aim of improving psychological or social wellbeing. Preliminary studies involving young people have shown benefits for wellbeing, mental health, and the development of social networks (Bertotti et al., 2020). Consequently, SP schemes could be explored further to enhance flourishing.

This study has several strengths, including the large sample, with follow-up of participants over up to 10 years and repeated administration of well-validated measures. Fixed effects models accounted for observed and unobserved individual heterogeneity. Our results were robust to adjustment for demographic, socioeconomic, and health-related time-varying confounders and different model specifications. Although we cannot make causal conclusions from observational data, arts engagement was longitudinally associated with subsequent flourishing. Future research should provide more causal evidence for the association between arts engagement and flourishing, such as in large randomized controlled trials or in a natural experiment with the introduction of SP in the USA.

This study also has limitations. Arts engagement was broadly defined, including anything that participants perceived as artistic, musical, or theatrical organized activities, making it difficult to identify the exact mechanisms through which engagement could influence flourishing. We also could not differentiate whether these activities were engaged in at school or college or outside of education, which may have different policy implications, as there are increased social gradients in out-of-school arts activities (Mak & Fancourt, 2021). Moreover, the categorization of arts engagement frequency was arguably arbitrary. Future studies may benefit from using a continuous measure of time and considering more specific types of arts engagement. Additionally, art, music, or theatrical activities are just a few of the varied cultural and creative activities young adults may have been participating in, such as literature, poetry, dance, and film. Many young people are also likely to have been taking classes in the arts and humanities at high school or college, but this also was not assessed in the TAS. We were thus unable to account for or measure the impact of these other forms of arts engagement on flourishing. Similarly, our conceptualization of flourishing was driven by the measures included in the TAS (Keyes, 2006b), and included a maximum of six questions on each domain of flourishing. It thus did not provide an in-depth assessment of wellbeing (Lee et al., 2021). Furthermore, although we adjusted for a range of demographic, socioeconomic, and health-related time-varying confounders, residual confounding is still possible. Unmeasured factors may influence both arts engagement and flourishing, including school environment and experiences, and other aspects of social integration, such as religious service attendance and community participation. We also recognize that gender is not a binary construct even though we had to treat it as such. We used an overly simple race/ethnicity variable (White, Black, Other), due to small numbers in Other groups, which conflates experiences across diverse groups. Future research should include more diverse samples and collect detailed data on race/ethnicity.

Considering other factors that contribute to flourishing is also a significant issue in this area of research. The ability to reach one’s potential depends not just on internal factors but also on formal political, economic, legal, and educational systems and structures, as well as patterns of injustice, structural racism, and historical trauma (Ryff & Kim, 2020; Willen et al., 2022). Our analyses focused specifically on change within individuals over time, meaning they accounted for individual heterogeneity in income, educational attainment, race/ethnicity, and other systemic factors that may influence flourishing. This is an important step towards understanding how arts engagement may influence flourishing independent of these factors. However, future research should further explore how the circumstances in which people live come together to determine both who is likely to participate in the arts and how such participation might influence their wellbeing over time.

In this study, increasing time spent doing artistic, musical, or theatrical organized activities was associated with enhanced flourishing in young people. Engaging in these activities at least weekly may be necessary to influence subsequent flourishing. The association with flourishing was driven primarily by increases in social wellbeing, accompanied by smaller increases in psychological wellbeing. These findings held across many subgroups of the population, indicating that all young people have the potential to benefit from the arts. However, those in non-metropolitan areas may be affected by fewer opportunities for engagement. Our findings also suggest that evidence from older adults may not be replicated in younger groups, highlighting the importance of future research that focuses on emerging adults. Considering ways to promote and facilitate participation in the arts could help to promote flourishing, enabling young people to lead healthier and more satisfying lives. This is particularly important given that flourishing is likely to not only be a product of arts engagement but also to contribute to future health-related behaviors.

## Supplementary Information

ESM 1 (PDF 258 kb)

## Additional Information

### Data Availability

This study used public data from the Transition into Adulthood Supplement of the Panel Study of Income Dynamics (PSID), which is available from the PSID website: https://simba.isr.umich.edu/data/data.aspx. Instructions to access the data are included with the analysis code. The analyses in this study were not preregistered.

### Code availability

Analysis code for the project can be obtained here: https://osf.io/8nbxd.
