# Supplementary material for: Longitudinal Associations Between Arts Engagement and Flourishing in Young Adults: A Fixed Effects Analysis of the Panel Study of Income Dynamics
Source: Affect Sci. 2022 Oct 11;4(1):131–42. doi: 10.1007/s42761-022-00133-6 (PMC10104994; doi:10.1007/s42761-022-00133-6)
Supplement: Supplementary file 1 — (PDF 258 kb) [file 42761_2022_133_MOESM1_ESM.pdf]

Supplementary Materials

Response rates

Table S1. *Response rates across waves of the Transition into Adulthood Supplement (TAS).*

Wave	Eligible for the TAS	Completed the TAS	Response rate
2005	839	745	89%
2007	1312	1115	85%
2009	1797	1559	87%
2011	2083	1907	92%
2013	2122	1804	85%
2015	1887	1641	87%
2017	2938	2526	86%
2019	3019	2595	86%

Languishing-Flourishing Scale

Table S2. *Languishing-Flourishing Scale items and domains.*

	"In the last month, how often did you feel…?"	Domain
		Emotional
1	Happy	Happiness
2	Interested in life	Interest
3	Satisfied	Life satisfaction
		Social
4	That you had something important to contribute to society	Contribution
5	That you belonged to a community like a social group, your school, or your neighborhood	Integration
6	That our society is becoming a better place	Actualization
7	That people are basically good	Acceptance
8	That the way our society works made sense to you	Coherence
		Psychological
9	Good at managing the responsibilities of your daily life	Mastery
10	That you have warm and trusting relationships with other people	Positive relations
11	That you have experiences that challenged you to grow or become a better person	Personal growth
12	Confident to think or express your own ideas and opinions	Autonomy
13	That you liked your personality	Self-acceptance
14	That your life had a direction or purpose	Purpose in life

Note. The response options for each item were: 1 = never, 2 = once or twice in the last month, 3 = about once a week, 4 = two or three times a week, 5 = almost every day, 6 = every day.

Descriptive statistics

Table S3. *Summary statistics for overall flourishing and each domain of flourishing.*

	Flourishing		Emotional wellbeing		Psychological wellbeing		Social wellbeing	
**Variation**	Mean	SD	Mean	SD	Mean	SD	Mean	SD
Overall	13.56	2.54	5.00	0.95	5.09	0.93	3.54	1.23
Between individual		2.08		0.74		0.72		0.97
Within individual		1.53		0.62		0.61		0.78

Note. Total flourishing scores could range from 1 to 18, and scores on each domain could range from 1 to 6.

Table S4. *Cross-tabulation between arts engagement and each outcome at baseline.*

	Flourishing	Emotional wellbeing	Psychological wellbeing	Social wellbeing
Arts engagement frequency				
Never	13.52 (2.54)	5.01 (0.94)	5.10 (0.93)	3.46 (1.27)
Monthly	13.90 (2.14)	5.08 (0.79)	5.15 (0.85)	3.73 (1.05)
Weekly	13.94 (2.34)	5.08 (0.88)	5.19 (0.84)	3.76 (1.17)
Daily	14.25 (2.50)	5.10 (0.93)	5.28 (0.83)	3.88 (1.23)
Overall	13.65 (2.49)	5.03 (0.92)	5.13 (0.91)	3.54 (1.25)

Note. Total flourishing scores could range from 1 to 18, and scores on each domain could range from 1 to 6.

Moderation by residential area

Table S5. *Main fixed effects models testing the longitudinal associations between arts engagement and flourishing presented separately according to the residential area of adolescents' family home.*

Arts engagement	Metropolitan area[a]				Non-metropolitan area[a]			
	Unadjusted		Adjusted		Unadjusted		Adjusted	
	Coef (95% CI)	p	Coef (95% CI)	p	Coef (95% CI)	p	Coef (95% CI)	p
Monthly	**0.19 (0.01 to 0.37)**	**0.040**	**0.19 (0.01 to 0.37)**	**0.039**	-0.05 (-0.34 to 0.25)	0.746	-0.06 (-0.36 to 0.23)	0.673
Weekly	**0.43 (0.24 to 0.62)**	**<0.001**	**0.41 (0.22 to 0.60)**	**<0.001**	0.01 (-0.27 to 0.29)	0.958	-0.04 (-0.32 to 0.24)	0.766
Daily	**0.60 (0.32 to 0.88)**	**<0.001**	**0.55 (0.27 to 0.83)**	**<0.001**	0.33 (-0.08 to 0.75)	0.111	0.21 (-0.21 to 0.62)	0.323

Note. N=3,328 (11,860 observations). Reference category in all models was never. Bold text indicates p<0.05.

a. Time-varying confounders were age, marital status, education, employment, family income, general health, and emotional/psychiatric problems.

Table S6. F*ixed effects models testing the longitudinal associations between arts engagement and flourishing presented separately according to the residential area of adolescents' family home, limited only to those who reported that they were still living with their parents.*

Arts engagement	Metropolitan area[a]				Non-metropolitan area[a]			
	Unadjusted		Adjusted		Unadjusted		Adjusted	
	Coef (95% CI)	p	Coef (95% CI)	p	Coef (95% CI)	p	Coef (95% CI)	p
Monthly	**0.29 (0.01 to 0.59)**	**0.040**	0.27 (-0.01 to 0.56)	0.060	0.18 (-0.36 to 0.72)	0.507	0.10 (-0.44 to 0.65)	0.713
Weekly	**0.33 (0.05 to 0.61)**	**0.020**	**0.31 (0.03 to 0.58)**	**0.032**	0.16 (-0.26 to 0.58)	0.451	0.04 (-0.40 to 0.48)	0.856
Daily	0.34 (-0.04 to 0.73)	0.087	0.26 (-0.12 to 0.65)	0.178	0.15 (-0.50 to 0.80)	0.645	0.11 (-0.58 to 0.80)	0.752

Note. N=2,858 (6,425 observations). Reference category in all models was never. Bold text indicates p<0.05.

a. Time-varying confounders were age, marital status, education, employment, family income, general health, and emotional/psychiatric problems.

Alternate model specifications

Table S7. *Arellano-Bond models using the difference GMM estimator.*

Arts engagement	Unadjusted		Adjusted[a]	
	Coef (95% CI)	p value	Coef (95% CI)	p value
**Model 1: Flourishing**				
Monthly	0.68 (-0.85 to 2.21)	0.384	-0.12 (-0.89 to 0.65)	0.761
Weekly	1.15 (-0.05 to 2.34)	0.060	0.56 (-0.27 to 1.38)	0.185
Daily	**1.59 (0.14 to 3.04)**	**0.032**	0.54 (-0.59 to 1.66)	0.350
**Model 2: Emotional wellbeing**				
Monthly	-0.30 (-0.94 to 0.33)	0.349	-0.10 (-0.43 to 0.23)	0.540
Weekly	0.46 (-0.01 to 0.93)	0.057	0.12 (-0.20 to 0.44)	0.449
Daily	-0.10 (-0.63 to 0.43)	0.714	-0.09 (-0.51 to 0.33)	0.668
**Model 3: Psychological wellbeing**				
Monthly	0.15 (-0.45 to 0.74)	0.629	0.01 (-0.30 to 0.32)	0.956
Weekly	-0.01 (-0.47 to 0.44)	0.949	0.06 (-0.25 to 0.37)	0.706
Daily	0.43 (-0.15 to 1.02)	0.143	0.24 (-0.20 to 0.69)	0.284
**Model 4: Social wellbeing**				
Monthly	0.48 (-0.24 to 1.20)	0.193	-0.03 (-0.42 to 0.36)	0.880
Weekly	0.52 (-0.17 to 1.20)	0.139	0.17 (-0.29 to 0.63)	0.471
Daily	**1.04 (0.22 to 1.86)**	**0.013**	0.40 (-0.30 to 1.10)	0.258

Note. N=2,128 (4,714 observations). Reference category in all models was never. Bold text indicates p<0.05.

a. Time-varying confounders were age, marital status, education, employment, family income, general health, and emotional/psychiatric problems.

Table S8. *Arellano-Bond models using the system GMM estimator (as in the main analyses) and limited to two lags.*

Arts engagement	Unadjusted		Adjusted[a]	
	Coef (95% CI)	p value	Coef (95% CI)	p value
**Model 1: Flourishing**				
Monthly	0.20 (-1.23 to 1.64)	0.781	-0.38 (-1.40 to 0.65)	0.471
Weekly	0.77 (-0.47 to 2.01)	0.222	0.78 (-0.22 to 1.79)	0.126
Daily	1.05 (-0.21 to 2.30)	0.102	0.53 (-0.64 to 1.70)	0.378
**Model 2: Emotional wellbeing**				
Monthly	0.06 (-0.55 to 0.67)	0.848	-0.02 (-0.38 to 0.34)	0.926
Weekly	0.08 (-0.41 to 0.57)	0.746	0.11 (-0.25 to 0.48)	0.538
Daily	0.08 (-0.43 to 0.59)	0.756	-0.08 (-0.51 to 0.34)	0.706
**Model 3: Psychological wellbeing**				
Monthly	-0.23 (-0.74 to 0.29)	0.390	-0.24 (-0.61 to 0.12)	0.194
Weekly	0.01 (-0.45 to 0.46)	0.969	0.04 (-0.32 to 0.41)	0.813
Daily	0.32 (-0.16 to 0.80)	0.196	0.12 (-0.36 to 0.60)	0.627
**Model 4: Social wellbeing**				
Monthly	0.33 (-0.31 to 0.96)	0.316	0.13 (-0.37 to 0.63)	0.610
Weekly	0.60 (-0.05 to 1.24)	0.069	0.45 (-0.08 to 0.98)	0.093
Daily	**0.91 (0.21 to 1.62)**	**0.011**	**0.78 (0.12 to 1.44)**	**0.020**

Note. N=3,175 (7,955 observations). Reference category in all models was never. Bold text indicates p<0.05.

a. Time-varying confounders were age, marital status, education, employment, family income, general health, and emotional/psychiatric problems.
